# Therapeutic Yoga Enhances Neuroplasticity and Metabolic Regulation Through Elevated Plasma Brain-Derived Neurotrophic Factor (BDNF) and Ghrelin in a Heterogeneous Cancer Survivor Population

**DOI:** 10.1177/15347354251385573

**Published:** 2025-10-23

**Authors:** Minal A. Sonawane, Alice K. Lee, Sagar Gaikwad, Gustavo J. Almeida, Nydia T. Darby, Tim Calderon, Amelie G. Ramirez, Daniel C. Hughes, Darpan I. Patel

**Affiliations:** 1 University of Texas Medical Branch, Galveston, TX, USA; 2 University of Texas Health Science Center at San Antonio, San Antonio, TX, USA; 3 The Open Hand Institute, San Antonio, TX, USA

**Keywords:** cognitive impairment, metabolic biomarkers, therapeutic yoga protocol

## Abstract

**Background:**

Cancer-related cognitive impairment (CRCI) frequently arises as a consequence of cancer treatments, manifesting in challenges such as impaired memory, attention, processing speed, and word finding. These cognitive deficits ranging from mild to moderate, can persist for months or even years. They can negatively impact a survivor’s quality of life (QOL), mental health, and interpersonal relationships. Moreover, cancer and its therapies adversely affect various metabolic processes in the body influencing factors such as weight changes, fat metabolism, energy regulation, dyslipidemia, growth hormone regulation, and cardiovascular health. A secondary analysis was conducted to investigate whether a 16-week therapeutic yoga program (TYP) modulates the cognitive and metabolic biomarkers profile in plasma among heterogeneous cancer survivors.

**Approach:**

Participants included in the study were adults aged 18 years and older with a clinical cancer diagnosis. Informed consent was obtained from all participants. Nineteen participants completed 3 weekly 75-minute sessions of TYP combined with love and kindness meditation. Blood samples were collected from 16 participants both before and after the TYP intervention. Eight neurological, metabolic, and inflammatory biomarkers (β-NGF, BDNF, Ghrelin, IL12P70, Leptin, MCP-1, TNF-β, VEGF-A) were measured by a U-Plex Custom Metabolic Group1 (hu) Multiplex Assay on the MESO Quickplex SQ 120MM, Model 1300. Data was analyzed using the Wilcoxon signed-rank test.

**Results:**

The participants had a mean age of 59.6 years (±7.3). Over half of the cohort (56%) were classified as overweight or obese (BMI ≥ 25 kg/m²). The majority were female (71%) and breast cancer survivors (65%), with 44% of these survivors being of Hispanic ethnicity. Statistically significant increases were observed in the concentrations of brain-derived neurotrophic factor (BDNF; pre: 653.50 vs post: 1234.17 pg/ml; 88.85% increase, *P* = .005) and ghrelin (pre: 576.10 vs post: 710.80 pg/ml; 23.38% increase, *P* = .04). Notably, a marked difference was found in vascular endothelial growth factor A (VEGF-A), which increased by 45.51% post-TYP, and monocyte chemoattractant protein-1 (MCP-1) decreased by 19.79% post-TYP.

**Conclusion:**

TYP contributed to substantial improvements in plasma levels of BDNF and ghrelin in this heterogeneous cohort of cancer survivors. Future research involving larger cohorts is needed to validate these findings.

## Background

The American Association of Cancer Research (AACR) Cancer Progress Report 2024 highlights remarkable strides in reducing cancer mortality, with a 33% decline in U.S. cancer death rates between 1991 and 2021, saving over 4.1 million lives. Advances in clinical oncology, including early detection and innovative treatments, have significantly extended survival for many patients.^1^ However, these achievements mask an enduring truth: cancer continues to impose a substantial burden, both as a disease and through the side effects of its treatments.

While treatments like chemotherapy and radiation improve survival, they often leave survivors grappling with debilitating side effects, including cognitive deficits collectively known as cancer-related cognitive impairment (CRCI). This condition impacts memory, attention, reasoning, and other critical cognitive functions. CRCI arises from the cancer itself or its treatments and is further exacerbated by factors such as age, genetics, and psychological well-being.^2^ Beyond cognition, treatments disrupt metabolic processes, influencing weight regulation, appetite homeostasis and energy regulation, dyslipidemia, growth hormone regulation, and cardiovascular health. Chronic inflammation, driven by pro-inflammatory cytokines like tumor necrosis factors (TNF) and interleukins, not only contributes to treatment side effects but also fuels cancer progression.^3,4^ Therefore, addressing the burden of cancer treatment side effects requires innovative approaches.

Exercise has long been recognized as a therapeutic intervention for improving physical and psychological outcomes in cancer survivors. However, cancer survivors often struggle with fatigue and prolonged recovery periods, limiting adherence to high-intensity exercise regimens. In this context, yoga, an ancient Indian practice integrating physical postures (asanas), controlled breathing techniques (pranayama), and meditation (dhyana), offers a promising, gentle, and effective intervention. Research indicates that therapeutic yoga significantly enhances survivor’s quality of life by improving cognitive, metabolic, physical, and emotional well-being.^5,6^ It effectively alleviates common side effects of cancer therapy, such as fatigue, pain, and emotional distress, while positively influencing markers of stress and recovery.^7^

Yoga has been shown to elevate serum levels of the neuroplasticity marker BDNF^8^ and reduce pro-inflammatory cytokines like TNF-α.^9^ Additionally, long-term yoga practice has been linked to increased circulating ghrelin levels, which regulate appetite and energy balance.^10^ For cancer survivors, particularly those with advanced metastatic disease, yoga has demonstrated benefits such as reduced fatigue, improved sleep quality, physical function^11^ and lower symptom burden.^12^ These findings align with evidence suggesting that yoga may modulate immune responses by reducing pro-inflammatory markers and enhancing anti-inflammatory pathways.^13^ Yoga’s anti-inflammatory effects may also stem from its influence on the autonomic nervous system. Practices such as pranayama have been shown to increase parasympathetic activity and decrease sympathetic overdrive, creating a favorable autonomic balance.^14,15^ Specifically, slow breathing exercises can significantly impact autonomic regulation, promoting relaxation and reducing chronic inflammation.^16^ Previously our group had found therapeutic yoga to modulate cytokines associated with cancer recurrence significantly.^17^

**Figure 1.**
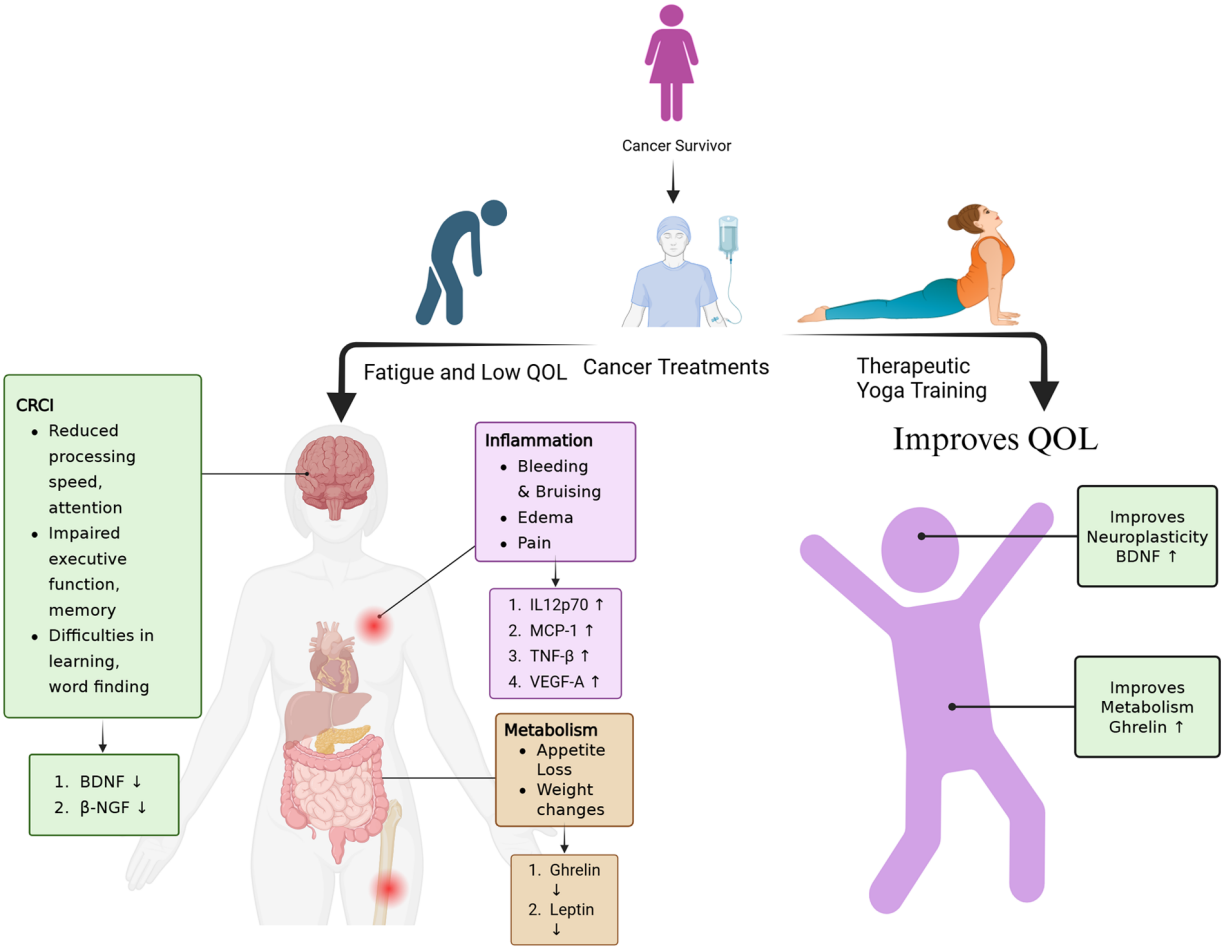
Impact of cancer treatments on cognitive, inflammatory, and metabolic health and the role of therapeutic yoga in enhancing quality of life (QOL). This figure illustrates the detrimental effects of cancer treatments on survivors, including cancer-related cognitive impairment (CRCI), inflammation, and metabolic dysregulation, leading to fatigue and reduced QOL. Therapeutic yoga, incorporating physical postures, breathing exercises, and meditation, mitigates these side effects by enhancing neuroplasticity (eg, increasing BDNF levels), reducing inflammation, and improving metabolic markers, promoting better physical, cognitive, and emotional health.

These insights highlight yoga’s potential as a therapeutic intervention to address both the physical and psychological side effects of cancer treatment. However, the extent to which yoga alleviates treatment-induced complications remains inadequately explored. To bridge this gap, we conducted a secondary analysis to evaluate the impact of a 16-week therapeutic yoga intervention on cognitive biomarkers like brain-derived neurotrophic factor (BDNF) and Nerve growth factor beta (β-NGF), metabolic markers (Leptin and Ghrelin), and inflammatory cytokines like monocyte chemoattractant protein-1 (MCP-1), tumor necrosis factor-beta (TNF-β), vascular endothelial growth factor-A (VEGF-A), and interleukin-12p70 (IL-12p70) in a heterogeneous cohort of cancer survivors. As illustrated in Figure 1, cancer treatments often lead to fatigue, inflammation, cognitive impairment, and metabolic dysregulation, collectively contributing to a decline in quality of life (QOL).^18-21^

Our findings revealed a significant increase in BDNF and Ghrelin levels following the yoga intervention, supporting the hypothesis that yoga may be an effective adjunct therapy to improve cognitive function, metabolic health, and inflammatory responses in cancer survivors.

## Methods

### Study Design and Subject Enrollment

This study utilized a single-arm, self-controlled, block enrollment design. Patient recruitment was conducted from January 2020 to March 2020. Participants were recruited from the local community through advertisements and word-of-mouth referrals. Flyers were distributed throughout the University of Texas Health San Antonio’s (UTHSA) Mays Cancer Center.

Interested individuals underwent an eligibility screening. The inclusion criteria required participants to be at least 18 years old, have a history of any cancer diagnosis (either currently undergoing treatment or post-treatment), possess a mobile phone or computer for survey completion and text communication, be able to speak and understand English or Spanish and be oriented to time and place. Exclusion criteria included enrollment in a competing protocol or any absolute contraindication to exercise testing as per the American College of Sports Medicine Guidelines on Exercise Testing and Exercise Prescription.^22^ Upon establishing contact with the potential participants, each was assigned a unique subject identification number. The participants were given a scripted brief explanation of the study in their language of choice (English or Spanish). Participants were informed that the study would commence once the target cohort size of 30 participants was achieved. A list of 43 interested participants was collected. In numerical order, the first 30 participants who passed screening for eligibility were invited to participate in the study and were scheduled for baseline assessments at the Holistic Exercise Advancement Laboratory (HEAL) at the UTHSA Mays Cancer Center. Out of the 30 invited, 29 provided informed consent and completed baseline assessments. Seventeen participants completed the intervention and contributed both baseline and post-intervention biospecimens. However, 1 participant was excluded from the biomarker analysis due to an insufficient sample volume.

### Demographics and Patient Characteristics

After consenting to the study, height, weight, blood pressure, demographic information, and medical history were collected. The body mass index was calculated as weight [kg]/(height [m]).^2^

### Description of Intervention

Participants were enrolled in a structured 16-week therapeutic yoga intervention conducted at the Mays Cancer Center, UTHSA as described previously.^17^ The intervention included 3 sessions per week, each lasting 75 minutes, and followed a meticulously designed therapeutic yoga protocol (TYP). This protocol incorporated yoga postures (asanas), controlled breathing exercises (pranayama), and loving-kindness meditation (LKM). A certified yoga instructor designed the TYP which has been described earlier.^23^ The traditional postures were thoughtfully modified to accommodate the physical abilities and limitations of the participants. The TYP was tailored to improve thoracic and extremity mobility, build muscular strength and endurance, enhance functional movement, guide safe transitions between the floor and standing positions, and alleviate stress. Additionally, it aimed to cultivate focus and mindfulness through breath-centered movements and introduce meditation practices, including LKM, during the final restorative phase.

Each TYP session followed a structured progression. Yoga postures were held for 3 slow, controlled breaths allowing participants to assess their comfort and tolerance while centering their focus on breathing. Sessions began with an introduction to traditional yoga practice followed by practicing Therapeutic Sun Salutation- a modified version of the traditional sequence accommodating individuals with balance impairments, joint restrictions, or limited flexibility—to ensure safety and accessibility. This was followed by smooth transitions through various sequences, including quadruped and prone poses, standing postures, seated movements, and restorative supine positions, culminating in the practice of LKM.

In response to the COVID-19 pandemic, the program shifted from an in-person to a virtual format after the first week to prioritize the health and safety of this high-risk population. The virtual protocol utilized the BlueJay Telehealth platform (Pleasanton, CA), allowing participants to access live-streamed sessions by asynchronous video recordings. Unique login credentials were provided to each participant to facilitate seamless access to the platform, ensuring continuity of the intervention despite pandemic-related disruptions.

### Cognitive, Metabolic, and Inflammatory Biomarkers Assessment

Blood samples were collected at baseline and at the end of the study using K_2_-EDTA tubes. Following collection, samples were allowed to rest at room temperature for 30 minutes before being centrifuged at 2000*g* for 15 minutes at 4°C to isolate plasma. The separated plasma was then aliquoted and stored at −80°C until biomarker analysis. Each sample was analyzed in duplicate using the Meso Scale Discovery U-Plex platform (MESO QuickPlex SQ 120MM) with the U-Plex Custom Human Metabolic Group 1 multiplex assay (K151ACM-1) Panel, which includes BDNF, β-NGF, Ghrelin Total, IL-12p70, Leptin, MCP-1, TNF-β, and VEGF-A. Multiplex analysis was meticulously conducted following the guidelines provided by MSD field scientists, within the biobehavioral laboratory at the University of Texas Medical Branch’s Schools of Nursing.

### Statistical Analysis

Study data were collected and managed using the REDCap electronic data capture tools hosted at UTHSA.^24^ REDCap (Research Electronic Data Capture) is a secure, web-based software platform designed to support data capture for research studies, providing (1) an intuitive interface for validated data capture; (2) audit trails for tracking data manipulation and export procedures; (3) automated export procedures for seamless data downloads to common statistical packages; and (4) procedures for data integration and interoperability with external sources. Per-protocol analysis was employed, and only those who completed the study protocol were included.

Within-group differences were evaluated using the Wilcoxon rank-sum test. Data are presented as median (interquartile range). The significance level was set at *P* < .05. The primary outcome was changes in cytokine concentrations after the completion of the 16-week TYP. The analysis was restricted to only the individuals that provided both baseline and end-of-study samples. All analyses were conducted using GraphPad Prism version 9.3.1 (San Diego, CA).

## Results

### Participant Demographics and Clinical Characteristics

**Figure 2.**
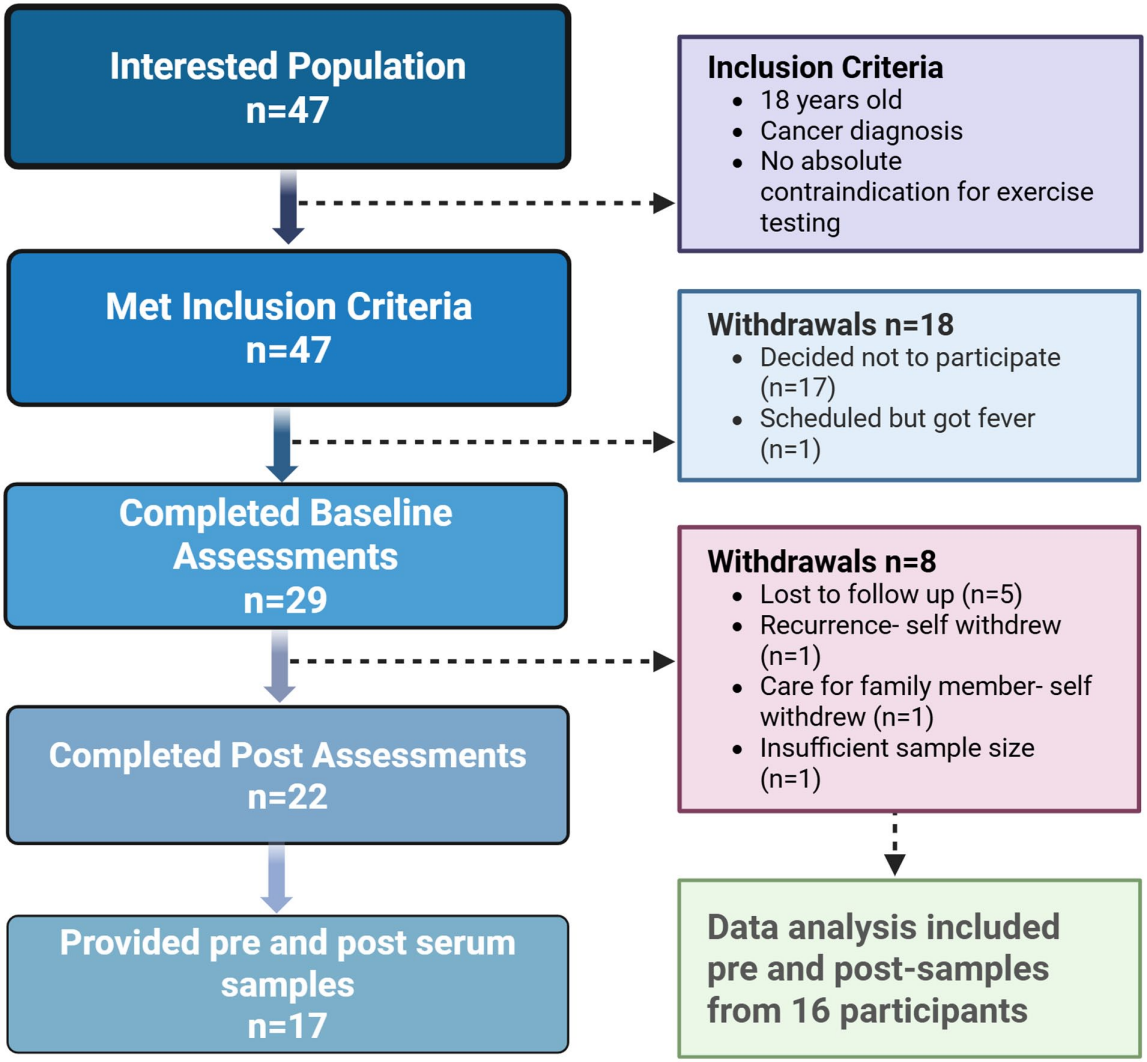
Flow diagram illustrating participant recruitment, retention, and sample inclusion for the study. A total of 47 individuals initially expressed interest and met inclusion criteria, out of which 29 completed baseline assessments. Subsequent withdrawals (n = 7) due to follow-up loss, personal or medical reasons, and insufficient sample availability led to 22 participants completing post-intervention assessments. Ultimately, matched pre- and post-intervention serum samples were available for 17 participants, with data from 16 included in the final analysis.

Of the 29 participants who consented, 22 completed the end-of-study visit, and 17 provided both baseline and end-of-study blood samples for biomarker analysis (Figure 2). One patient was removed from the analysis due to insufficient sample volume, resulting in a total of 16 patients included in the biomarker analysis. The participants had a mean age of 59.1 ± 7.3 years (range: 47-69) and were predominantly overweight or obese (69% with BMI ≥ 25 kg/m²). The majority were female (81%), breast cancer survivors (68%). Eight of the 16 participants identified as Hispanic. Detailed demographic information is provided in the Supplemental File. The remaining 6 participants were not included due to issues such as hemolysis, insufficient sample volume, or reluctance to provide additional blood.

### Cognitive, Metabolic, and Inflammatory Markers Response to Therapeutic Yoga

A statistically significant increase in plasma levels of BDNF and ghrelin was observed in cancer survivors following a 16-week yoga intervention, indicating potential therapeutic benefits of yoga in this population. Table 1 highlights changes in concentrations of the biomarkers assessed in this study. Data is represented as pg/ml.

**Table 1.** Changes in Neurotrophic, Metabolic, and Inflammatory Biomarkers Following Therapeutic Yoga Intervention in Cancer Survivors.

Neuroinflammatory proteins	Pre median (IQR)	Post median (IQR)	Δ (%)	*P*-value
β-NGF	0.6898 (1.3098, 1.9996)	1.0341 (1.3459, 2.3801)	49.92	.2312
BDNF	**645.98 (162.62, 808.61)**	**1136.95 (1534.62, 397.67)**	**76.00**	**.0052**
IL-12p70	0.4803 (0.2422, 0.7226)	0.4966 (0.2262, 0.7229)	3.41	.9799
MCP-1	75.05 (131.98, 207.03)	78.68 (195.68, 116.99)	4.84	.2312
VEGF-A	4.798 (7.848, 12.64)	8.817 (8.437, 17.25)	83.75	.5896
TNF-β	0.097 (3.63, 3.72)	0.217 (3.84, 3.62)	123.16	.4037
Metabolic proteins				
Ghrelin	**436.18 (373.48, 809.66)**	**439.27 (400.77, 840.05)**	**0.71**	**.0413**
Leptin	23 609.93 (11 218.66, 34 828.6)	22 154.7 (10 677.89, 32 832.6)	−6.16	.7820

This table highlights pre- and post-intervention median values, interquartile ranges (IQR), percent change (∆), and statistical significance (*P*-value) for key biomarkers. Therapeutic yoga demonstrated a significant increase in brain-derived neurotrophic factor (BDNF, *P* = .0052) and ghrelin (*P* = .0413) levels, suggesting improved neuroplasticity and metabolic regulation. The bold entries in Table 1 represent statistically significant differences (*P* < 0.05) between pre- and post-intervention values.

## Discussion

The purpose of this secondary analysis was to evaluate whether the TYP—an intervention previously shown to reduce pro-tumorigenic cytokines in cancer survivors^17^—also significantly alters plasma levels of a panel of cognitive, metabolic and inflammatory biomarkers within the same cohort. Our findings support the hypothesis that TYP has a positive influence on biomarkers linked to cognitive and metabolic functions in cancer survivors. Notably, we observed significant increases in plasma BDNF and ghrelin levels, suggesting potential therapeutic benefits.

BDNF is a critical growth factor in the central nervous system, playing a key role in neural plasticity and neurogenesis.^25^ Upregulation of BDNF has been associated with improved memory function and a reduced risk of neurodegenerative diseases such as Alzheimer’s disease.^26^ Conversely, decreased BDNF levels have been linked to neuropsychiatric disorders, including major depressive disorder (MDD) and Alzheimer’s disease.^27^ BDNF signaling plays a critical role in neurogenesis, synaptic plasticity, learning, and memory, with physical activity and cognitive engagement serving as key modulators of this pathway.^28^ Given that cognitive function and synaptic plasticity are directly influenced by BDNF levels,^29-31^ and that BDNF signaling is often diminished in neurodegenerative and psychiatric disorders,^25,32^ understanding the mechanisms underlying BDNF modulation is crucial.

Elevated BDNF levels following exercise-based lifestyle interventions have been associated with reduced neuronal apoptosis and demyelination.^33,34^ Consistent with our findings, a randomized controlled trial by Tolahunase et al demonstrated that a 12-week yoga and meditation-based lifestyle intervention (YMLI) significantly increased BDNF levels in a cohort of 58 MDD patients. This increase was strongly correlated with reduced depression severity, highlighting the therapeutic potential of yoga in enhancing neuroplasticity.^35^ Similarly, Cartmel et al^36^ reported an increase in total BDNF levels following a 6-month aerobic exercise intervention in ovarian cancer survivors. However, our study did not differentiate between free and total BDNF, warranting further investigation into specific BDNF fractions and their functional relevance.

The increase in BDNF observed in this study suggests a potential link between yoga practices—including postures, breathing exercises, and meditation—and neurotrophic signaling. Previous research has established that both physical and neuronal activity significantly upregulate BDNF gene expression in the brain,^37,38^ leading to the activation of signaling pathways that enhance learning and memory through exercise-dependent mechanisms.^31^ Future research should explore the specific contributions of yoga components to BDNF regulation, including its distinct fractions and underlying mechanistic pathways, to better understand its role in cognitive recovery among cancer survivors.^39^

Our findings showed a slight, though not statistically significant, increase in β-NGF levels, indicating a potential role of TYP in modulating neurotrophic signaling. β-NGF is a critical neurotrophic factor that supports the development, maintenance, and survival of peripheral and central cholinergic neurons. NGF plays a protective role in experimental models of neuronal injury and age-related cholinergic decline, making it a promising therapeutic target for AD.^40^

Although evidence on the effect of mind-body interventions on NGF levels in individuals with cognitive impairment is limited, a pilot study by Balasubramanian et al^41^ demonstrated that acute yogic breathing (YB) practice significantly increased salivary NGF levels in 60% of participants, compared to no changes in the active control group. Additionally, previous studies have shown that intranasally administered NGF can bypass the blood-brain barrier, reaching the spinal cord within 24 hours.^42^ These findings suggest that stimulating endogenous NGF expression through mind-body practices like YB may exert neuroprotective effects on cholinergic neurons. Although the β-NGF increase in our study did not reach statistical significance, the observed trend highlights the potential of TYP in promoting neurotrophic signaling and warrants further exploration in larger studies.

The results of our study also indicated a significant increase in the metabolic protein ghrelin. Ghrelin, often referred to as the “hunger hormone,” plays a crucial role in metabolism and appetite regulation. Several studies have demonstrated that ghrelin levels tend to increase in response to exercise and dietary interventions, particularly in the context of weight loss. For instance, a randomized trial by Puklin et al,^43^ and findings by Kim et al^44^ reported elevated ghrelin levels following such interventions.

A systematic review by Ouerghi et al^45^ highlighted that acute forms of physical activity, including short-term, resistance, and intermittent exercise, did not significantly alter ghrelin levels. However, more than half of the studies examining chronic and higher-intensity exercise reported an increase in circulating ghrelin. Our findings align with these observations, as we observed a statistically significant increase in ghrelin levels post-intervention.

This increase in ghrelin may have important implications for cancer survivors. Elevated ghrelin levels have been associated with improved appetite, weight gain, and the inhibition of pro-inflammatory cytokine production.^46^ Given the role of chronic inflammation in cancer progression, the potential anti-inflammatory effects of ghrelin warrant further investigation. Future studies should explore the long-term impact of exercise-induced ghrelin elevation on cancer outcomes to better understand its therapeutic potential in survivorship and metabolic health.

Most participants exhibited a decrease in leptin levels following the 16-week yoga intervention, but it was not statistically significant. Leptin, an adipokine primarily secreted by adipose tissue, plays a key role in appetite regulation and energy homeostasis.^47^ Elevated leptin levels have been associated with increased tumor growth and metastasis, as leptin can promote cell proliferation, angiogenesis, and inflammation within the tumor microenvironment.^48,49^ The impact of physical exercise on leptin levels remains a topic of debate. While some studies suggest that reductions in leptin concentrations are influenced by factors such as exercise duration and caloric expenditure,^50-52^ others have found no significant changes following physical activity.^53^

We did not see a statistically significant change in any of the inflammatory biomarkers, and age may play a role in this variable. Franceschi et al^54^ showed that the natural aging process is associated with increased inflammation. Given that most of our subjects were older than 60 years old, this may have played a role in the increased trends shown in all 3 of our inflammatory biomarkers. Our yoga intervention is similar to a randomized control trial by Bower et al,^55^ where they found that their 12-week Iyengar-based yoga intervention decreased pro-inflammatory gene expression in breast cancer survivors. They evaluated plasma levels of receptors for pro-inflammatory cytokines, such as sTNF-RII, to measure the activity of the corresponding cytokine, and these generally remained stable in the yoga group.

Although the reduction in plasma MCP-1 levels following the 16-week TYP was not statistically significant, we observed a downward trend, suggesting yoga’s potential role in modulating inflammation. Physical exercise has been widely associated with reduced systemic inflammation.^56^ Notably, endurance exercise has been shown to decrease MCP-1 levels in individuals with metabolic syndrome,^57^ while even modest physical activity has been reported to lower circulating inflammatory markers, including MCP-1, in patients with heart failure.^58^ Given that yoga incorporates both physical movement and stress reduction, it may contribute to anti-inflammatory effects through mechanisms similar to those observed in other forms of exercise. We believe this study provides preliminary evidence supporting the need for further investigation with a larger cohort to validate yoga’s effect on reducing MCP-1 plasma levels. While this study provides evidence for the role of TYP in modulating neuroprotective and metabolic proteins, it is not without limitation. Due to the COVID-19 pandemic, the majority of yoga sessions were delivered virtually. While this allowed continued participation, virtual delivery may have reduced participant engagement, adherence, or instructor feedback, potentially influencing intervention efficacy. Additionally, the relatively small sample size limits the statistical power of our findings, emphasizing the need for larger, well-powered studies to validate these results and enhance their generalizability. Consequently, some of the non-significant findings (eg, VEGF-A, MCP-1) may be attributable to Type II errors. Another important limitation is the predominance of female breast cancer survivors and Hispanic participants in our cohort, which may limit the generalizability of the observed effects of yoga to the broader population of cancer survivors. While the TYP incorporated both static and dynamic components with modifications for varying physical abilities, outcomes were not stratified by baseline physical function. Future studies should explore whether physical capability or yoga modality differentially influences biomarker responses. Additionally, potential confounders such as diet, physical activity outside the intervention, and concurrent therapies were not controlled, warranting more rigorous monitoring in future research. Furthermore, the absence of cognitive function assessments in our clinical trial restricts the depth of interpretation regarding biomarker changes. Additionally, the absence of a control group limits the ability to distinguish the observed effects from natural recovery, placebo effects, or other confounding variables. Future randomized controlled studies are needed to confirm the efficacy of yoga interventions.

This pilot study provides preliminary evidence that therapeutic yoga may positively influence neuroplasticity and metabolic regulation in cancer survivors, as indicated by increased plasma BDNF and ghrelin levels. Despite the limitations, our results have implications for the lifestyle modifications and recommendations that physicians can make for cancer patients’ post-chemotherapy to help prevent the known cognitive and metabolic decline. Future studies with statistically powered samples can confirm the trends seen in this pilot study for all the non-statistically significant changes in cognitive, metabolic, and inflammatory biomarkers.

## Supplemental Material

sj-pdf-1-ict-10.1177_15347354251385573 – Supplemental material for Therapeutic Yoga Enhances Neuroplasticity and Metabolic Regulation Through Elevated Plasma Brain-Derived Neurotrophic Factor (BDNF) and Ghrelin in a Heterogeneous Cancer Survivor Population Supplemental material, sj-pdf-1-ict-10.1177_15347354251385573 for Therapeutic Yoga Enhances Neuroplasticity and Metabolic Regulation Through Elevated Plasma Brain-Derived Neurotrophic Factor (BDNF) and Ghrelin in a Heterogeneous Cancer Survivor Population by Minal A. Sonawane, Alice K. Lee, Sagar Gaikwad, Gustavo J. Almeida, Nydia T. Darby, Tim Calderon, Amelie G. Ramirez, Daniel C. Hughes and Darpan I. Patel in Integrative Cancer Therapies
