# Supplementary material for: Therapeutic Yoga Enhances Neuroplasticity and Metabolic Regulation Through Elevated Plasma Brain-Derived Neurotrophic Factor (BDNF) and Ghrelin in a Heterogeneous Cancer Survivor Population
Source: Integr Cancer Ther. 2025 Oct 23;24:15347354251385573. doi: 10.1177/15347354251385573 (PMC12559641; doi:10.1177/15347354251385573)
Supplement: sj-pdf-1-ict-10.1177_15347354251385573 – Supplemental material for Therapeutic Yoga Enhances Neuroplasticity and Metabolic Regulation Through Elevated Plasma Brain-Derived Neurotrophic Factor (BDNF) and Ghrelin in a Heterogeneous Cancer Survivor Population [file sj-pdf-1-ict-10.1177_15347354251385573.pdf]

Pt/Record ID	Age	Cancer Type	Years of Survivorship	Gender	Ethnicity	Race	BMI	BMI Classification	BDNF pg/ml		β-NGF pg/ml		Ghrelin pg/ml		Leptin pg/ml		MCP-1 pg/ml		TNFβ pg/ml		VEGF-A pg/ml		IL-12p70 pg/ml	
									Pre	Post	Pre	Post	Pre	Post	Pre	Post	Pre	Post	Pre	Post	Pre	Post	Pre	Post
0637-001	51	Breast	4	Female	Not Hispanic or Latina	Caucasian	30.48	Obese	377.87	396.74	2.31	1.95	1078.00	885.18	35751.52	43360.70	194.96	252.15	3.72	3.69	10.69	10.73	0.26	0.37
0637-002	68	Non-Hodgkin's Lymphoma	7	Female	Hispanic or Latina	Caucasian	30.28	Obese	183.23	811.399978	1.21	1.25	493.61	620.20	50306.34	30900.49	152.41	131.63	3.64	3.64	7.72	7.60	0.20	0.21
0637-003	64	Prostate	10	Male	Not Hispanic or Latina	Caucasian	30.99	Obese	140.25	42.43	1.84	1.07	452.88	419.93	31908.49	12191.77	198.06	105.07	3.67	3.63	11.22	10.15	0.60	0.35
0637-006	66	Breast	20	Female	Hispanic or Latina	Hispanic	28.88	Overweight	84.39	500.14	1.40	1.33	473.75	785.60	34520.96	38628.91	159.10	101.84	3.76	3.71	6.99	8.25	0.90	0.83
0637-007	48	Breast	8	Female	Hispanic or Latina	Hispanic	25.24	Overweight	1097.49	2553.42	1.97	2.37	814.73	1400.03	17122.77	16750.90	125.54	200.50	4.07	4.27	37.93	73.41	2.18	2.86
0637-008	62	Breast	8	Male	Not Hispanic or Latina	Caucasian	32	Obese	170.09	1760.45	1.85	2.60	392.81	501.67	12480.46	11538.80	132.82	198.94	3.66	3.66	9.78	16.45	0.03	0.05
0637-010	49	Breast	4	Female	Not Hispanic or Latina	Asian	23.04	Normal/healthy	2504.11	4317.69	1.02	1.06	760.81	825.01	10890.27	12509.86	119.77	194.59	3.59	3.62	12.26	19.66	0.09	0.21
0637-013	65	Breast	28	Female	Hispanic or Latina	Hispanic	29.58	Overweight	514.38	933.26	5.04	5.10	807.98	797.19	22485.87	23953.59	197.12	168.08	4.92	4.83	7.89	7.74	0.56	0.61
0637-019	55	Breast	1	Female	Not Hispanic or Latina	African American	23.59	Normal/healthy	318.88	1276.47	1.31	1.35	372.05	350.38	11946.51	6565.72	185.65	134.89	3.57	3.58	16.82	50.66	0.07	0.23
0637-021	63	Chronic lymphocytic leukemia	2	Male	Hispanic or Latina	Hispanic	29.18	Overweight	423.73	13.17	1.30	1.45	337.88	388.68	7441.06	4572.85	129.45	119.86	3.66	3.67	7.53	6.76	0.66	0.61
0637-022	58	Epithelial, Sarcoma & Thyroid	5	Male	Hispanic or Latina	Hispanic	31.11	Obese	85.82	26.14	2.08	2.40	373.96	541.38	7496.61	9816.99	818.15	108.41	3.65	4.19	13.81	9.39	0.96	3.57
0637-023	62	Breast	1	Female	Not Hispanic or Latina	Caucasian	25.43	Overweight	1952.26	3236.84	125.25	134.81	212881.88	21288187.78	83446.16	78446.44	2926.68	2567.60	6.16	6.63	236.03	342.58	1645.26	1556.65
0637-025	65	Breast	15	Female	Not Hispanic or Latina	Caucasian	23.87	Normal/healthy	171.03	397.98	1.73	1.90	368.52	400.47	16302.69	17913.18	233.06	145.48	3.67	3.68	8.41	8.50	0.47	0.29
0637-026	57	Breast	2	Female	Hispanic or Latina	Hispanic	41.53	Obese	1630.16	1459.35	1.84	1.71	331.93	356.53	52709.72	66557.96	206.83	168.30	3.59	3.57	11.66	11.56	0.31	0.14
0637-027	68	Thyroid, Basal Cell Carcinoma	9	Female	Not Hispanic or Latina	Caucasian	19.47	Normal/healthy	90.12	973.12	1.12	1.52	1739.70	2699.75	843.70	1339.32	70.20	95.29	3.61	3.61	7.52	13.44	0.54	0.69
0637-029	66	Breast	4	Female	Hispanic or Latina	Hispanic	22.86	Normal/healthy	712.32	1048.17	1.93	1.80	419.02	400.88	11328.13	19939.81	207.62	165.64	3.67	3.73	12.26	12.13	0.42	0.35

BDNF - Brain Derived Neurotrophic Factor
β-NGF - Beta Nerve Growth Factor
MCP-1 - Monocyte Chemoattractant Protein-1
TNFβ - Tumor Necrosis Factor Beta
VEGF-A - Vascular Endothelial Growth Factor A
IL-12p70 - Interleukin 12 p70 subunit
